# Global analysis of gene expression and projection target correlations in the mouse brain

**DOI:** 10.1007/s40708-015-0014-2

**Published:** 2015-03-20

**Authors:** Ahmed Fakhry, Tao Zeng, Hanchuan Peng, Shuiwang Ji

**Affiliations:** 1 Department of Computer Science, Old Dominion University, Norfolk, VA 23529 USA; 2 Allen Institute for Brain Science, Seattle, WA 98103 USA

**Keywords:** Projection targets, Gene expression patterns, Visualization, Clustering, Correlation, Feature selection

## Abstract

Recent studies have shown that projection targets in the mouse neocortex are correlated with their gene expression patterns. However, a brain-wide quantitative analysis of the relationship between voxel genetic composition and their projection targets is lacking to date. Here we extended those studies to perform a global, integrative analysis of gene expression and projection target correlations in the mouse brain. By using the Allen Brain Atlas data, we analyzed the relationship between gene expression and projection targets. We first visualized and clustered the two data sets separately and showed that they both exhibit strong spatial autocorrelation. Building upon this initial analysis, we conducted an integrative correlation analysis of the two data sets while correcting for their spatial autocorrelation. This resulted in a correlation of 0.19 with significant *p* value. We further identified the top genes responsible for this correlation using two greedy gene ranking techniques. Using only the top genes identified by those techniques, we recomputed the correlation between these two data sets. This led to correlation values up to 0.49 with significant *p* values. Our results illustrated that although the target specificity of neurons is in fact complex and diverse, yet they are strongly affected by their genetic and molecular compositions.

**Electronic supplementary material:**

The online version of this article (doi:10.1007/s40708-015-0014-2) contains supplementary material, which is available to authorized users.

## Introduction

The functions of neurons are largely determined by their molecular compositions. Those molecules are encoded by the genome that is expressed uniquely in each neuron. The mammalian brain contains a large number of neurons that are connected in diverse patterns, resulting in complex interaction networks that control information flow. In those interaction networks, neurons typically have very diverse projection target specificity. For example, projections from the primary somatosensory cortex (S1) target both cortical and subcortical regions [1]. Also, it has been shown that cortico-cortical projections in the mouse visual cortex are also functionally target specific [2]. To obtain a better understanding of the diversity of projection neuron classes, transcriptome analysis of the neurons along with a direct correlation with projection targets is needed.

The integrative analysis of neuronal gene expression and connectivity patterns was initially carried on the worm *Caenorhabditis elegans* as its gene expression and neuron-level connectivity are simple and largely known [3–6]. Those studies showed that the genetic properties of neurons significantly influence their synaptic network structures. Kaufman et al. [4] performed a co-variation correlation experiment known as Mantel test and illustrated that gene expression and connectivity patterns are significantly correlated. A similar analysis was performed later on the mammalian brain, leading to more significant results [7–9]. Specifically, French and Pavlidis [8] carried out a large-scale analysis of the transcriptome-connectome correlation in the rodent brain, leading to a correlation of 0.25. These high correlations inspired other studies to even predict the connectome based on the gene expression patterns. Wolf et al. [10] performed this prediction with an accuracy up to 83 % in the rodent brain. In addition, they identified many genes that contribute most to this high prediction. Similarly, Ji et al. [11] obtained a very high prediction accuracy of 93 % by using the Allen Brain Atlas data. They were able to achieve almost the same accuracy when using a few number of most predictive genes. Such analysis has recently been extended to the human brain [12].

The abovementioned studies focused on analyzing how the gene expression patterns of source and target neurons are correlated as compared to neurons that are not connected. The prediction studies used the expression patterns of target neurons to predict their connectivity with a particular source neuron. On the other hand, increasing evidence has shown that there are also direct correlations between source neuron gene expression patterns and projection target specificity [13]. In a recent study, efforts have been made to identify genes that are expressed in specific excitatory projection neuron classes [1]. The study showed that the neocortex contains diverse populations of excitatory neurons that are definable by their specific cortical and subcortical projection targets. However, some of the most broadly used markers for specific layers were found not to be expressed selectively in neurons with a specific projection target. This indicates that in spite of the significant correlations between marker genes and projection targets, the excitatory neuron projection targets are in fact diverse and complex [1].

In this study, we conducted in a global, quantitative analysis of gene expression and projection target correlations in the adult mouse brain. We mainly focused on studying how the gene expression patterns in the source neurons are globally related to projection target specificity. In this sense, our study is fundamentally different from the prior ones reported in [8–11]. Instead, our work was mainly motivated by [1] and aimed at a global, quantitative analysis that is lacking to date. By using the Allen Mouse Brain Atlas and the Allen Mouse Brain Connectivity Atlas data, we started by visualizing and clustering the injection site gene expression patterns and projection targets separately. These initial analyses showed that both data sets exhibit strong spatial autocorrelation. That is, nearby injection sites tend to express similar sets of genes and also tend to project to similar targets.

To account for spatial autocorrelation, we performed the partial Mantel test [14] in which the spatial effect is corrected. We found that even after correcting for the spatial autocorrelation, the two data sets are highly correlated with a partial correlation of 0.19. We adopted two greedy gene ranking approaches to identify the top genes responsible for this correlation. Using only the top genes identified by our gene ranking techniques in the correlation analysis, we were able to obtain a series of significant correlations with values up to 0.49. These results indicate that the voxel gene expressions directly affect their target projections. These results are consistent with the findings reported in [1], but have extended the previous study to a global and quantitative analysis.

## Material and methods

In our experiments, we used two data sets from the Allen Brain Atlas (ABA) [15]. Specifically, we used data from the Allen Mouse Brain Atlas [16] and the Allen Mouse Brain Connectivity Atlas [17], which provide gene expression data and connectivity data, respectively, in the adult mouse brain. To allow an integrated study of both data sets, the ABA provides an annotated 3D reference model upon which all images from both atlases were aligned. Both atlases provide grid-level voxel data obtained from images mapped to the same 3D reference space.

### Allen Mouse Brain Atlas

The Allen Mouse Brain Atlas (the Gene Expression Atlas) provides in situ hybridization (ISH) data in the male P56 C57BL/6J mouse brain. Genome-wide data are provided in sagittal sections, and coronal sections for about 4000 genes with restricted expression patterns are also provided. Our experiments were carried out on the coronal genes, since these include functionally important genes. When multiple data sets are available for the same gene, we computed the average values across data sets. For this atlas, the grid-level voxel data are provided at 200 μm resolution.

### Allen Mouse Brain Connectivity Atlas

In the Allen Mouse Brain Connectivity Atlas (the Connectivity Atlas), axonal projections in the mouse brain are visualized by viral tracers from more than 200 regions. This atlas provides axonal projections along with injection voxel coordinates for 1788 injection sites. We treated each injection data set independently throughout the experiments though some of the brain regions were injected multiple times, since the specific injection voxels are unique. In this atlas, the grid-level voxel data are provided at 100 μm resolution.

### Data extraction and processing

**Fig. 1:**
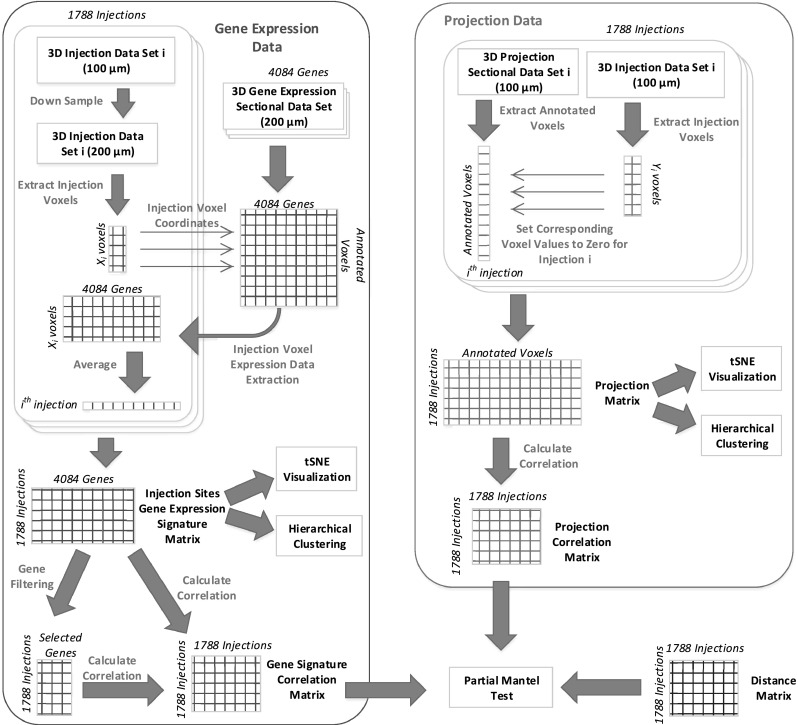
Illustration of the data extraction and processing pipeline. The *left* and *right* panels show the steps involved in processing the gene expression and connectivity data, respectively. The processed data were used along with a distance matrix to perform the partial Mantel test

To perform an integrative analysis of gene expression patterns and projection targets, the gene expression and connectivity data sets should be mapped to the same space as they are originally provided in different resolutions. The data extraction and processing steps are illustrated in Fig. 1. Specifically, the coronal gene expression data are provided for approximately 4000 genes in a 3D grid-level format at a 200 μm resolution. For each gene, we extracted the energy values at the 60,452 voxels annotated in the reference atlas. The extracted voxels for each gene form a column of the gene expression data matrix. The connectivity data are provided for 1788 injection sites at a 100 μm resolution. Similar to the gene data, we extracted the energy values at more than 4,00,000 annotated voxels from each projection data set corresponding to a specific injection site. Those extracted voxels form the columns of the projection data matrix. The two processed data sets were used later in our experiments to generate the injection sites gene correlation and projection correlation matrices.

To make an integrative analysis of the two data sets possible, the gene signature of each injection site is needed. We obtained the gene signature of each injection site by first down-sampling its injection voxels to the 200 μm resolution and then extracting the rows corresponding to those voxels from the gene signature matrix. The number of injection voxels is usually different for different injection sites. We computed the average gene signature across all injection voxels to come up with a vector of approximately 4000 genes representing the gene signature of a single injection site. This vector forms a column in the injection site gene signature matrix that was used later throughout our experiments. We observed that the energy values of the injection voxels are usually very high, as they represent injection values instead of projection energy. To eliminate these data artifact, we set the values of injection voxels to zero for each injection site independently.

### Data visualization using* t*-SNE

We intended to study the relationship between gene expression patterns and projection target specificity for different injection sites. To this end, we visualized the high-dimensional gene expression and projection target signatures associated with each injection site using the * t*-distributed stochastic neighbor embedding (* t*-SNE) method [18, 19]. * t*-SNE is an extension of SNE [20] to simplify the optimization and overcome the so-called “crowding problem”. * t*-SNE aims to model local structures of high-dimensional data points while ensuring that global dissimilarity between clusters is preserved. To this end, * t*-SNE computes two similarity matrices; one is obtained based on symmetrized Gaussian conditional distributions of original data space, and one is computed from Student * t*-distributions of low dimensional space. The low dimensional data, known as map points, is learned by minimizing the Kullback–Leibler (KL) divergence between the probability distributions in the original data space and the embedding space. Since KL divergence is not symmetric, different types of mismatches contribute differently to the overall cost. As a result, nearby map points are produced to represent nearby original data points, while distant map points are derived to reflect the original data points that are far apart. It has been shown that * t*-SNE is able to preserve the local structure of the high-dimensional data points, and its objective function is particularly straightforward to optimize in comparison to the original SNE objective [19]. * t*-SNE has been used in the visual exploration of high-dimensional gene expression data [21].

In the context of our experiments, we aimed at mapping the high-dimensional gene expression and connectivity data associated with each injection site to 2D space. For each injection site, we generated gene expression and projection target signature vectors representing the gene expression and projection targets for each of the 1788 injection sties. For the gene expression data, each vector contains 4084 elements that correspond to the gene expression values of the 4084 genes in the injection site. Similarly, each projection target vector contains 60,452 values representing the projection strength from the injection site to the 60,452 voxels in the entire brain. The gene expression and projection target vectors for all injection sites were collected into matrices, and * t*-SNE was applied to map these high-dimensional vectors onto 2D space for visual exploration.

### Hierarchical clustering

We employed hierarchical clustering to further explore the gene expression and projection target patterns. Hierarchical clustering constructs a dendrogram to represent the relations among all data points in a data set. Each leaf in the dendrogram represents an individual data point and each internal node represents a cluster. Such clustering method is particularly useful when the number of clusters is unknown. There are two common approaches for performing hierarchical clustering. The agglomerative approach begins by treating each individual data point as a cluster and successively merges cluster pairs with minimal inter-cluster distance. This process repeats until a single cluster containing all the data points is obtained. In contrast, the divisive approach starts from a single cluster containing the entire data set and recursively split each cluster until each data point forms a single cluster.

Two important parameters in hierarchical clustering are the similarity measure between two data points and the criteria for computing the inter-cluster similarity. Hierarchical clustering uses linkage criteria to compute inter-cluster similarity. Three commonly used linkage criteria are single, complete, and average linkages, which define similarity between two clusters as the minimum, maximum, and average similarity between members in two clusters, respectively. For distance metrics, the cosine, Person correlation and Spearman correlation are commonly applied in hierarchical clustering.

Given that brain structures are hierarchically organized based on morphology and function, we hypothesized that constructing hierarchical clusters from voxels that contain expression and connectivity information is likely to recover similar brain hierarchical ontology. To test this hypothesis, we used agglomerative hierarchical clustering with complete linkage and Pearson correlation to construct dendrogram for both the gene expression and the projection target data.

Specifically, each injection site is associated with a gene expression vector and a projection target vector. These vectors are treated as individual data points. The matrix containing all the injection site gene expression vectors was used in gene expression clustering while the matrix containing all the injection site projection vectors was used in connectivity clustering.

### Partial mantel test

We generated the gene expression correlation matrix and the projection target correlation matrix from the injection site gene expression and projection target data matrices, respectively. We are interested in studying the relationship between gene expression patterns and projection target correlations by integrating those two correlation matrices.

Mantel test [22] determines the statistical significance of the correlation between two correlation matrices, and is a tool that matches our need. Our experimental results indicate that both gene expression and project target are strongly correlated with spatial distance. That is, nearby injection sites tend to express similar sets of genes and also tend to project to similar targets. To account for spatial autocorrelation, we performed the partial Mantel test [14, 23] in which the spatial effect is excluded. Since both gene expression and projection target correlate significantly with the injection site physical distance, partial Mantel test becomes essential when studying their correlation together.

To perform partial Mantel tests, we generated a distance matrix capturing the pairwise distance between all injection sites. Specifically, we first computed the coordinate of each injection site by averaging the coordinates of all voxels belonging to that injection site. We then calculated the Euclidean distance between each pair of injections based on the averaged coordinates. We also tried using the log of the Euclidean distance, and this resulted in very similar results. The resulting distance matrix was used along with the gene correlation and projection correlation matrices to perform the partial Mantel test. This test determines the statistical significance of results by computing the *p* value. Specifically, the data were randomly permuted 1000 times and the *p* value is computed as the probability that the same or higher correlation value is achieved by the randomized data.

### Greedy group gene selection

The injection site gene correlation matrix described in Sect.  2.6 was computed based on the correlation of all genes in the coronal set. Since not all genes contribute equally to the correlation with projection targets, we employed greedy strategies to identify subsets of genes that correlate most with the projection targets. Essentially, we aimed at removing some columns of the injection site gene signature matrix before the correlation matrix was generated. We used two greedy techniques to obtain a gene ranking that can help eliminating the least important genes.

In the greedy group gene selection approach, we followed a greedy method used in [4]. This method operates in an iterative way. In each iteration, we computed a score for each gene as the Mantel test value after eliminating its corresponding column from the injection site gene signature matrix. This score indicates the importance of each gene in determining the correlation with projection targets. After the scores for all genes were computed, a specific percentage of the least important genes were then eliminated as a group from the data set before proceeding to the next iteration. This operation continued until a predefined number of genes were obtained.

To make the greedy approach more robust, this procedure was repeated multiple times using 50 % of the data randomly sampled from the original set each time. We then constructed a frequency vector for all the genes containing the frequency that each gene was selected among the multiple repetitions. Note that a similar approach was first used in [4], but the goal was not to obtain a gene ranking. We modified this technique and increased the number of repetitions and decreased the sampling percentage to obtain a gene frequency ranking. We refined our gene frequency ranking by combining the results generated from applying this procedure several times with different parameters. We used different numbers of repetitions, different stopping criteria.

### Greedy single gene selection

We also employed a greedy single gene selection approach as in [8] to obtain a complete gene ranking for all the genes used in our experiments. Similar to the group selection method, we computed a score for each gene in each iteration of the method to capture its effect on the correlation with the projection targets. In each iteration, only the least important gene was removed. This procedure continued until all genes were eliminated. By treating the gene that was removed first as the least important gene, we can obtain a complete gene ranking from this method. In comparison with the group selection method, the single gene selection method is much more computationally expensive. We used a parallel implementation for this scheme in order to accelerate the computation.

## Results and discussion

In this section, we report the results of visualizing the gene expression and connectivity target data by projecting them onto 2D space using * t*-SNE. We then performed hierarchical clustering on these two data sets to gain further insights. The primary aim of this work was to provide an integrative analysis of these two data sets and study their relationships.

### Gene expression and projection targets visualization

We used * t*-SNE to visualize the gene expression and projection target data. The gene expression matrix contains 1788 rows, and the columns represent all the genes. * t*-SNE was used to reduce the number of columns to 2, thereby facilitating data visualization. Similarly, the projection target matrix was also reduced to 2D. We associated each injection data set with its primary injection structure and used the same color code provided by the ABA for visualization. The ABA color code assigns each brain structure a unique color, where nearby structures are given similar colors. The color code used in visualization is provided in Supplemental Fig. 1 as in [24].

**Fig. 2:**
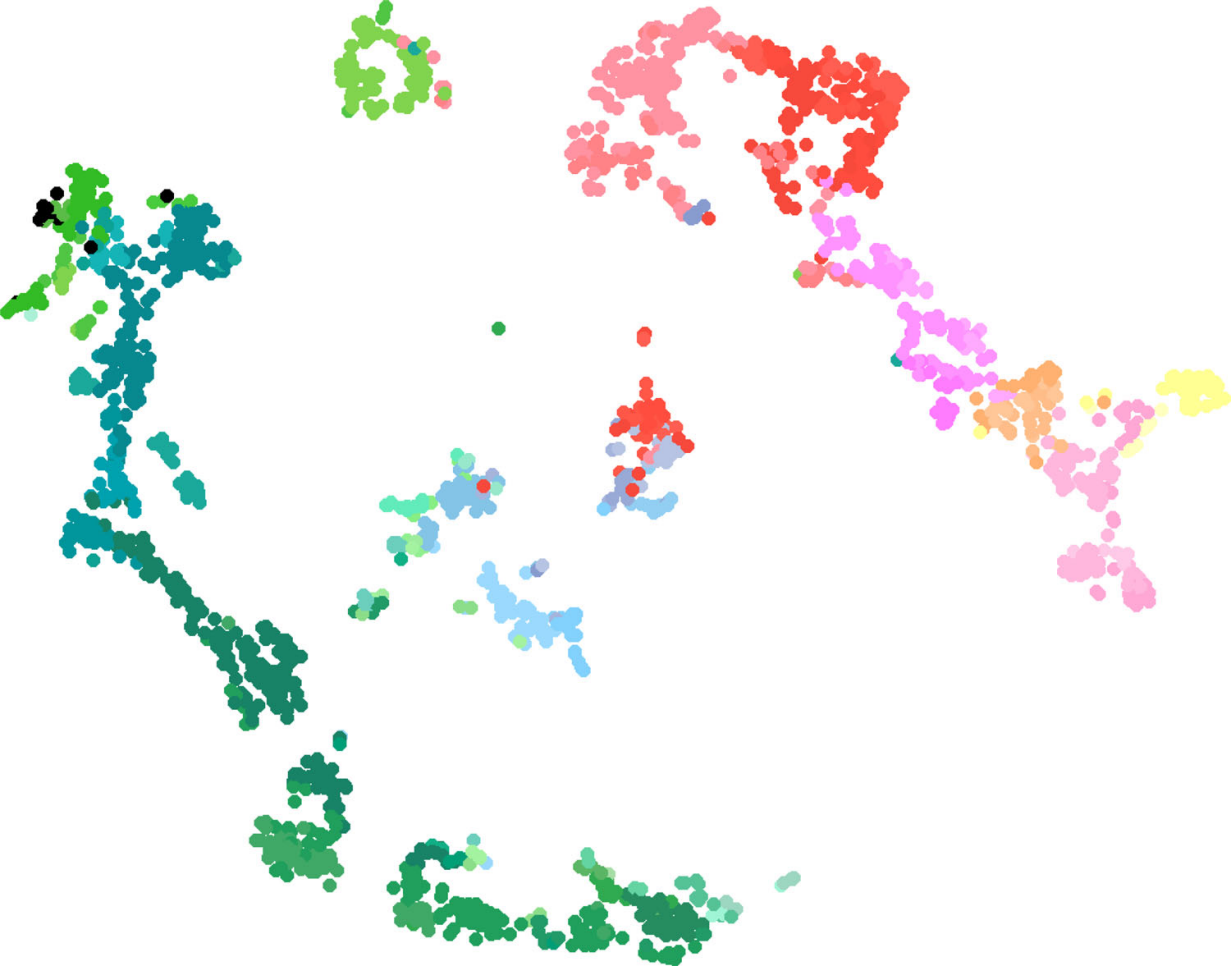
Scatter plot visualization of the injection site gene expression data after mapping to 2D space using * t*-SNE. Each injection site is associated with its primary injection structure. The *colors* of structures were obtained from the ABA, where similar *colors* represent related brain structures. The complete *color* code used in visualization is provided in Supplemental Fig. 1

The visualization of gene expression data is given in Fig. 2. We can observe that voxels with similar colors were mapped to nearby locations. This shows that gene expression patterns correlate strongly with spatial distance, a result consistent with prior findings [21, 25–28]. Specifically, voxels were mainly separated into two groups, namely the brain stem and the cerebrum. In brain stem, voxels of substructures of interbrain, midbrain and hindbrain were grouped together. In cerebrum four major groups were observed: visual cortex, sensory-motor cortices and the rest of cortex areas, cerebral nuclei and hippocampal formation.

**Fig. 3:**
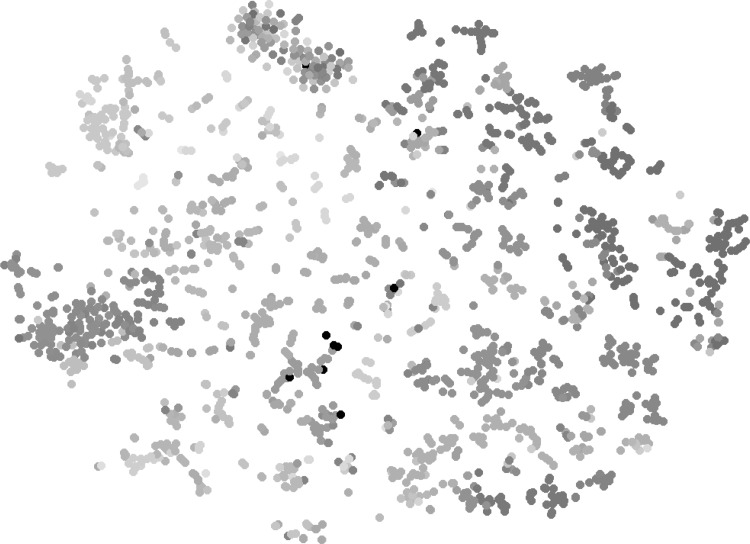
Scatter plot visualization of the injection site projection target data after mapping to 2D space using * t*-SNE. Each injection site is associated with its primary injection structure. The *colors* of structures were obtained from the ABA, where similar *colors* represent related brain structures. The complete *color* code used in visualization is provided in Supplemental Fig. 1

Unlike the gene expression data results, * t*-SNE visualization of the projection target data in Fig. 3 was unable to show clear boundaries between brain structures. Nevertheless, this result shows that interbrain, midbrain and hindbrain structures from brainstem were still largely preserved. Although cerebrum voxels were more scattered in 2D space in comparison to those of the brain stem, some spatial structures were observed for visual cortex, sensory-motor cortices and hippocampal formation. We also observed that some voxels from brain stem were mixed with those from cerebrum. This could reflect similar connectivity patterns between them due to their characteristics in terms of neuronal information processing. For example, thalamus relays information between subcortical nuclei and the cerebral cortex. Therefore, the connectivity of voxels from cerebral cortex remains similar to those of thalamus being connected to them.

Our results illustrate that the * t*-SNE projection of gene expression data showed a high consistency with the neuroanatomy. Similar colors representing nearby regions were mapped to nearby locations, forming clusters that are similar to the brain anatomy. This indicates that the gene expression data clearly demonstrate a strong spatial locality. A similar relationship also holds for the projection target data, but to a less extent. These results indicate that both gene expression and projection target patterns exhibit spatial locality with different levels of significance.

### Hierarchical clustering

We used the gene expression and connectivity data matrices directly in hierarchical clustering. The gene expression matrix contains 1788 rows representing all the injection sites and 4084 columns representing all the genes. Similarly, the connectivity data matrix contains 1788 injection data sets as rows and 60,452 brain voxels as columns. We used agglomerative hierarchical clustering with complete linkage and Pearson correlation as the similar measure on both data sets. The same color code provided by the ABA was used in the dendrogram.

**Fig. 4:**
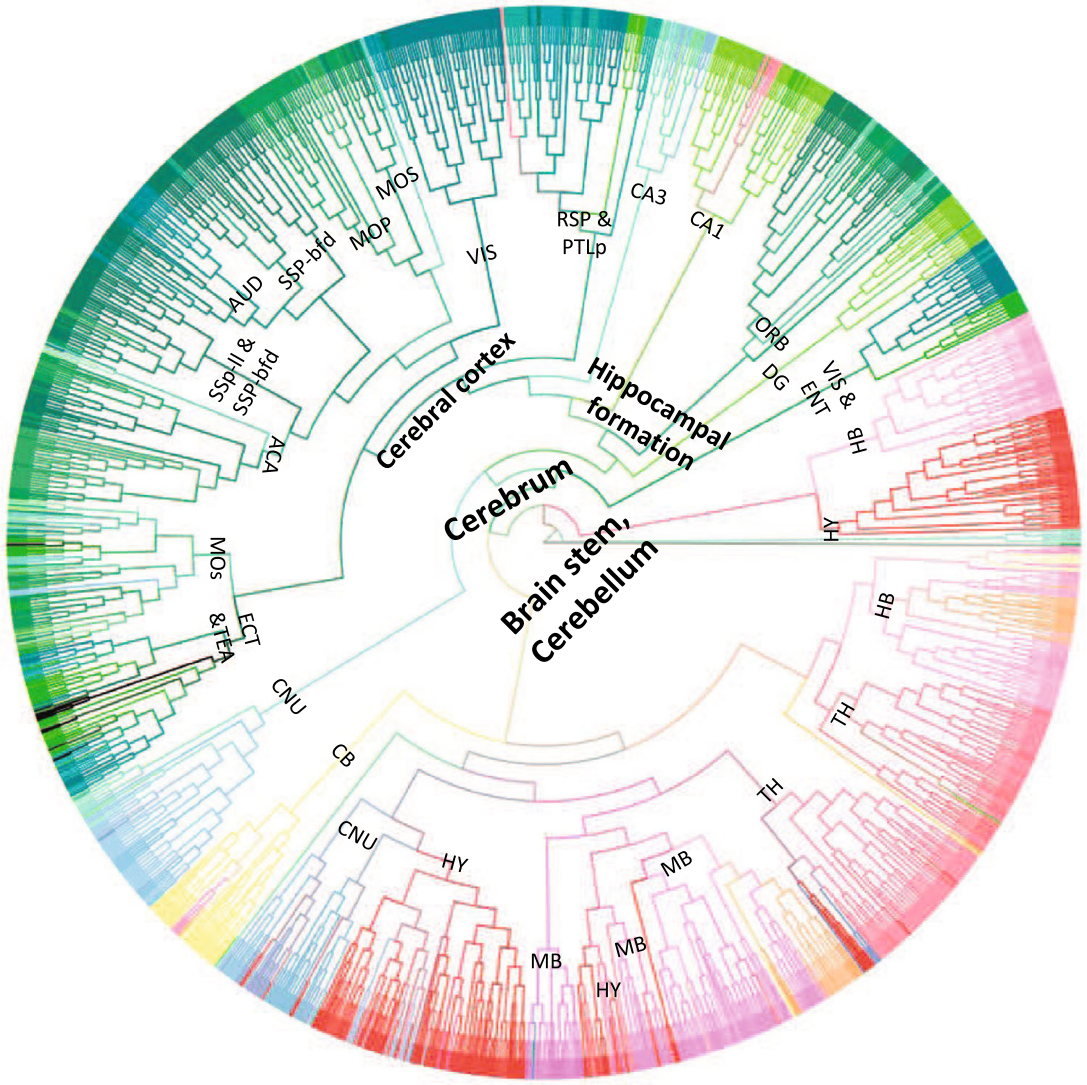
Dendrogram generated by hierarchical clustering on the injection site gene expression data. The acronyms annotated on the cluster nodes were given based on the brain structure that majority of voxels in the leaf node belong to. The *colors* of brain structure were obtained from the ABA, where similar *colors* represent related brain structures. The complete *color* code is provided in Supplemental Fig. 1. The acronyms and the corresponding full brain structure names are as follows: *ACA* anterior cingulate area, *AUD* auditory areas, *CA1* filed CA1, *CA3* field CA3, *CB* cerebellum, *CNU* cerebral nuclei, *DG* dentate gyrus, *ECT* ectorhinal area, *ENT* entorhinal area, *HB* hindbrain, *HY* hypothalamus, *MB* midbrain, *MOp* primary motor area, *MOs* secondary motor area, *ORB* orbital area, *RSP* retrosplenial area, *SSp-bfd* primary somatosensory area, barrel field, *SSP-ll* primary somatosensory area (lower limb), *TEa* temporal association areas, *TH* thalamus, *VIS* visual areas, *PTLp* posterior parietal association areas

Figure 4 shows the dendrogram for the gene expression data set. Similar to the * t*-SNE visualization result, hierarchical clustering on gene expression data resulted in two major clusters, namely the brain stem and cerebrum. The voxels of interbrain, midbrain and hindbrain largely form clusters. In the cerebrum, the clusters of visual cortex, sensory-motor cortices, auditory cortex and cerebral nuclei can be clearly observed.

**Fig. 5:**
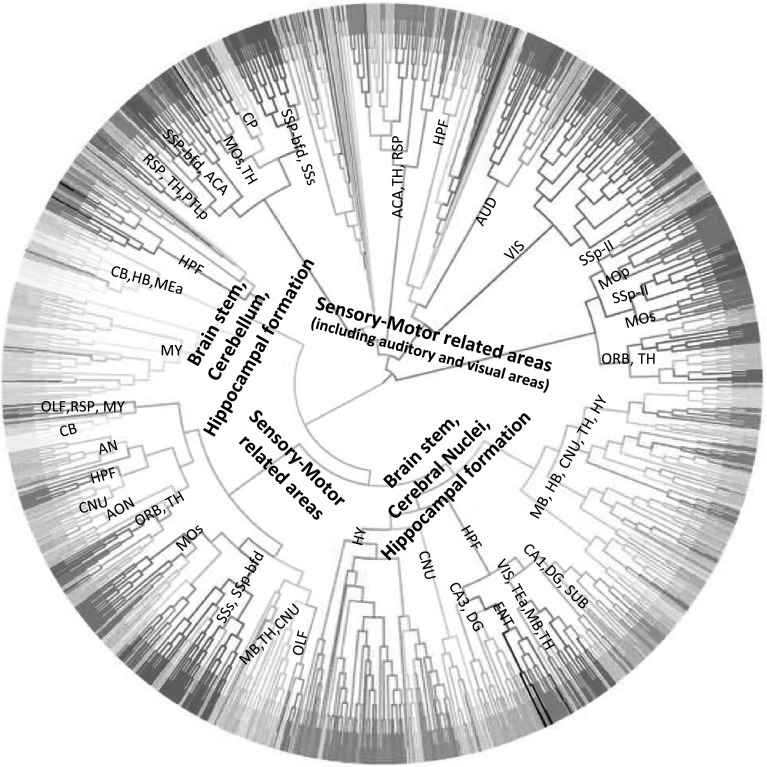
Dendrogram generated by hierarchical clustering on the projection target data. The acronyms annotated on the cluster nodes were given based on the brain structure that majority of voxels in the leaf node belong to. The *colors* of brain structure were obtained from the ABA, where similar *colors* represent related brain structures. The complete *color* code is provided in Supplemental Fig. 1. The acronyms and the corresponding full brain structure names are as follows: *ACA* anterior cingulate area, *AUD* auditory areas, *AN* amygdala nuclei, *CA1* filed CA1, *CA3* field CA3, *CB* cerebellum, *CNU* cerebral nuclei, *DG* dentate gyrus, *ENT* entorhinal area, *HB* hindbrain, *HY* hypothalamus, *MB* midbrain, *MOp* primary motor area, *MOs* secondary motor area, *OLF* olfactory areas, *ORB* orbital area, *RSP*retrosplenial area, *SSp-bfd* primary somatosensory area, barrel field, *SSP-ll* primary somatosensory area (lower limb), *TEa* temporal association areas, *TH* thalamus, *VIS* visual areas, *PTLp* posterior parietal association areas. *SUB* subiculum, *HPF* hippocampal formation, *CP* caudoputamen

Figure 5 shows the dendrogram for the projection target data set. We can observe that this clustering generated four major clusters. The first two clusters primarily involve in sensory-motor related functions, with one of which contains voxels belonging to visual and auditory area exclusively. For the other two clusters, in addition to both containing hippocampal formation and brain stem voxels, one includes cerebellum voxels and one contains cerebral nuclei voxels. In the cerebrum, we observed that despite the voxels of cerebellar cortex tend to cluster together based on their neuronal functions, they are mixed with voxels of other subcortical nuclei and the thalamus of interbrain. Such patterns revealed in our hierarchical clustering is consistent with known neuronal connectivity and function of the thalamus. That is, thalamus is heavily interconnected with subcortical nuclei and the cerebral cortex and plays an important role as information relay center. Hence, voxels of thalamus exhibit connectivity patterns similar to those of voxels from cerebellar cortex to which they are connected.

Overall, we observed that the clusters generated from the gene expression data were more consistent with the brain anatomy than the clusters generated from the connectivity data. These results are consistent with the results of visualization. Both experiments showed that spatial locality is stronger in the gene expression data than in the projection target data.

### Gene expression and projection target correlations

The primary aim of this study was to investigate the correlation between gene expression patterns and projection target specificity. By visualizing and clustering the gene expression and projection data sets, it is clear that both of them demonstrate spatial autocorrelation. We therefore employed the partial Mantel test to correlate these two data sets while the spatial effect is eliminated.

We constructed the injection site gene expression correlation matrix by computing the correlation between the rows of the gene signature matrix. Similarly, the injection site projection target correlation matrix is constructed by computing the correlation between the rows of the projection signature matrix. These two correlation matrices capture the correlations between gene expression patterns and projection target specificity in the same set of injection sites. To eliminate the spatial autocorrelation effect, we constructed a physical distance matrix that captures the pairwise Euclidean distance between the injection sites. Another distance matrix was constructed using the log of the Euclidean distance, and this resulted in very similar results. We performed partial Mantel test to quantify the significance of correlation between these two correlation matrices while eliminating their spatial autocorrelation. This test resulted in a correlation score of 0.1981 with a *p* value of less than 0.001. The significance of the correlation result indicates that the gene expression patterns and projection target specificity are significantly correlated, a result consistent with the previous findings [1].

**Fig. 6:**
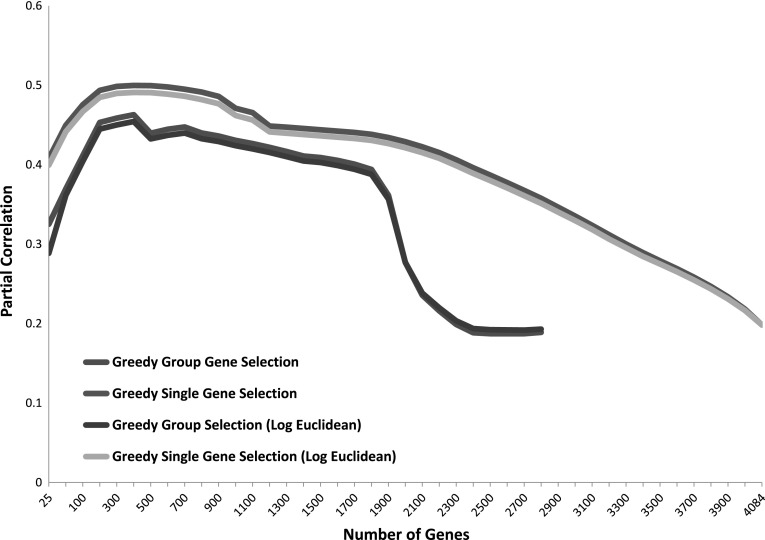
The partial Mantel test results obtained by using different numbers of top ranked genes generated from two greedy gene selection approaches (group gene selection and single gene selection). The group gene selection approach provides a ranking for approximately 2800 genes only. All the remaining genes are eliminated together at the same time, so they cannot be represented on the graph. The results of using the log of Euclidean distance are also shown

Motivated by previous studies [4, 8], we also tried to maximize the correlation score by selecting a subset of genes. Specifically, we used the greedy gene selection approaches to obtain a gene ranking and used different numbers of top ranked genes to compute the injection site gene correlation matrix. We used two greedy techniques to obtain gene rankings as described in the Material and Methods. The detailed results of the partial Mantel test corresponding to different numbers of top genes is shown in Fig. 6.

It is clear from the result that the partial Mantel correlation can be significantly improved when a subset of selected genes were used. The two gene selection methods yielded a single peak approximately when the top 400 genes were used in computing the gene expression correlation matrix. The correlation scores obtained by using the top 400 genes were 0.4998 and 0.4629 for the single and group selection techniques, respectively. These scores are much higher than the score obtained by using all the genes. We note that all the *p* values corresponding to the results in Fig. 6 are less than 0.001 and thus are significant. The result also shows that the single gene selection technique yielded higher correlation results than the group selection method. This is reasonable as the group selection method might exclude a batch of highly important and less important genes simultaneously as they had similar rankings at a specific iteration. On the other hand, the single gene selection technique re-evaluates all the remaining genes at every iteration after excluding one gene at a time. While both techniques had a single peak at approximately 400 genes, after closely examining those genes, we found that they only overlap in 89 genes which accounts for 22 % overlap. This indicates that the high correlation obtained is not attributed to individually important genes but rather to gene groups. The top 400 genes selected by each technique are provided in Supplemental Table 1.

## Conclusion

Our work represents the first global analysis of the gene expression and projection target correlations in the adult mouse brain. We studied each modality separately and revealed their own characteristics to set the stage for the integrative study. We showed through visualization and clustering that both the gene expression and the projection targets data demonstrated significant levels of spatial autocorrelation that needs to be accounted for in the integrative analysis. By using the partial Mantel test, we showed that these two modalities were significantly correlated even after correcting for spatial autocorrelation. We employed greedy gene selection technique and used it to generate gene rankings. Based on the gene ranking results, we obtained much higher correlations by using different numbers of the top genes. The correlations results reported in this study are more significant than the values reported in previous studies given that the spatial autocorrelation effect has been eliminated.

This study is one of the first studies towards exploring the correlation of gene expression patterns and projection target specificity at a brain-wide scale. Given that the gene expression and the projection targets are highly correlated, a lot more in-depth analysis in this area could be further pursued. We will explore different patterns of gene expression that result in specific projection target patterns in the future. We will also perform in-depth analysis on the top genes identified in this study and investigate their functions. We will investigate whether this type of correlation between gene expression patterns and projection target specificity holds in other brains such as the human brain.

## Electronic supplementary material

Below is the link to the electronic supplementary material.

Supplementary material 1 (pdf 87 KB)

Supplementary material 2 (xlsx 20 KB)
