# Supplementary material for: Global analysis of gene expression and projection target correlations in the mouse brain
Source: Brain Inform. 2015 Mar 20;2(2):107–17. doi: 10.1007/s40708-015-0014-2 (PMC4883149; doi:10.1007/s40708-015-0014-2)
Supplement: Supplementary file 1 — Supplementary material 1 (pdf 87 KB) [file 40708_2015_14_MOESM1_ESM.pdf]

Supplemental Figure 1: Color codes for the ARA brain structures

Abbreviation	Structure name
GU	Gustatory areas
PTLp	Posterior parietal association areas
AUDp	Primary auditory area
AUDd	Dorsal auditory area
AUDv	Ventral auditory area
AUD	Auditory areas
VISp	Primary visual area
VISam	Anteromedial visual area
VISal	Anterolateral visual area
VISl	Lateral visual area
VISpl	Posterolateral visual area
VISpm	posteromedial visual area
VISp4	Primary visual area, layer 4
ECT	Ectorhinal area
PERI	Perirhinal area
VISC	Visceral area
TEa	Temporal association areas
SSp–un	Primary somatosensory area, unassigned
SSp	Primary somatosensory area
SSp–bfd	Primary somatosensory area, barrel field
SSp–ll	Primary somatosensory area, lower limb
SSp–m	Primary somatosensory area, mouth
SSp–tr	Primary somatosensory area, trunk
SSp–ul	Primary somatosensory area, upper limb
SSs	Supplemental somatosensory area
RSP	Retrosplenial area
RSPd	Retrosplenial area, dorsal part
RSPv	Retrosplenial area, ventral part
RSPagl	Retrosplenial area, lateral agranular part
MOp	Primary motor area
MOs	Secondary motor area
AId	Agranular insular area, dorsal part
AIp	Agranular insular area, posterior part
AI	Agranular insular area
ORB	Orbital area
ORBl	Orbital area, lateral part
ORBm	Orbital area, medial part
ORBvl	Orbital area, ventrolateral part
PL	Prelimbic area
ENT	Entorhinal area
ENTl	Entorhinal area, lateral part
ENTm	Entorhinal area, medial part, dorsal zone
ACA	Anterior cingulate area
ACAd	Anterior cingulate area, dorsal part
ACAv	Anterior cingulate area, ventral part
POST	Postsubiculum
SUB	Subiculum
SUBd	Subiculum, dorsal part
SUBv	Subiculum, ventral part
AON	Anterior olfactory nucleus

Abbreviation	Structure name
ILA	Infralimbic area
PRE	Presubiculum
COApl	Cortical amygdalar area, posterior part, lateral zone
COApm	Cortical amygdalar area, posterior part, medial zone
TTd	Taenia tecta, dorsal part
PIR	Piriform area
PAR	Parasubiculum
CA1	Field CA1
CA3	Field CA3
DG	Dentate gyrus
IA	Intercalated amygdalar nucleus
AAA	Anterior amygdalar area
MEA	Medial amygdalar nucleus
MEAad	Medial amygdalar nucleus, anterodorsal part
MEAav	Medial amygdalar nucleus, anteroventral part
MEApd	Medial amygdalar nucleus, posterodorsal part
CEA	Central amygdalar nucleus
CEAc	Central amygdalar nucleus, capsular part
ACB	Nucleus accumbens
OT	Olfactory tubercle
BMA	Basomedial amygdalar nucleus
BMAp	Basomedial amygdalar nucleus, posterior part
GPe	Globus pallidus, external segment
GPi	Globus pallidus, internal segment
CLA	Claustrum
LS	Lateral septal nucleus
LSc	Lateral septal nucleus, caudal (caudodorsal) part
LSr	Lateral septal nucleus, rostral (rostroventral) part
LSv	Lateral septal nucleus, ventral part
SF	Septofimbrial nucleus
LA	Lateral amygdalar nucleus
NLOT	Nucleus of the lateral olfactory tract
MS	Medial septal nucleus
TRS	Triangular nucleus of septum
NDB	Diagonal band nucleus
CP	Caudoputamen
MOB	Main olfactory bulb
BLAa	Basolateral amygdalar nucleus, anterior part
AOB	Accessory olfactory bulb
EPd	Endopiriform nucleus, dorsal part
MA	Magnocellular nucleus
SI	Substantia innominata
DP	Dorsal peduncular area
TR	Postpiriform transition area
BAC	Bed nucleus of the anterior commissure
BST	Bed nuclei of the stria terminalis
LHA	Lateral hypothalamic area
LPO	Lateral preoptic area
PSTN	Parasubthalamic nucleus
STN	Subthalamic nucleus

Abbrev.	Structure name
TU	Tuberal nucleus
ZI	Zona incerta
TMv	Tuberomammillary nucleus, ventral part
PMv	Ventral premammillary nucleus
SUMl	Supramammillary nucleus, lateral part
MM	Medial mammillary nucleus
MPN	Medial preoptic nucleus
SUM	Supramammillary nucleus
VMH	Ventromedial hypothalamic nucleus
AHN	Anterior hypothalamic nucleus
PH	Posterior hypothalamic nucleus
PMd	Dorsal premammillary nucleus
PVp	Periventricular hypothalamic nucleus, posterior part
PVpo	Periventricular hypothalamic nucleus, preoptic part
AVPV	Anteroventral periventricular nucleus
SCH	Suprachiasmatic nucleus
MPO	Medial preoptic area
DMH	Dorsomedial nucleus of the hypothalamus
ARH	Arcuate hypothalamic nucleus
PVH	Paraventricular hypothalamic nucleus
SO	Supraoptic nucleus
SCs	Superior colliculus, sensory related
IC	Inferior colliculus
MEV	Midbrain trigeminal nucleus
NB	Nucleus of the brachium of the inferior colliculus
ICc	Inferior colliculus, central nucleus
ICd	Inferior colliculus, dorsal nucleus
ICe	Inferior colliculus, external nucleus
PBG	Parabigeminal nucleus
MGd	Medial geniculate complex, dorsal part
MGv	Medial geniculate complex, ventral part
LGd	Dorsal part of the lateral geniculate complex
SPFm	Subparafascicular nucleus, magnocellular part
SPFp	Subparafascicular nucleus, parvicellular part
MG	Medial geniculate complex
SPA	Subparafascicular area
VAL	Ventral anterior–lateral complex of the thalamus
VM	Ventral medial nucleus of the thalamus
VPL	Ventral posterolateral nucleus of the thalamus
VPM	Ventral posteromedial nucleus of the thalamus
VPMpc	Ventral posteromedial nucl. of thalamus, parvicellular part
PO	Posterior complex of the thalamus
POL	Posterior limiting nucleus of the thalamus
AMd	Anteromedial nucleus, dorsal part
IAD	Interanterodorsal nucleus of the thalamus
AM	Anteromedial nucleus
PVT	Paraventricular nucleus of the thalamus
PT	Parataenial nucleus
LD	Lateral dorsal nucleus of thalamus
LGv	Ventral part of the lateral geniculate complex

Abbreviation	Structure name
RE	Nucleus of reunions
LH	Lateral habenula
LP	Lateral posterior nucleus of the thalamus
AV	Anteroventral nucleus of thalamus
RT	Reticular nucleus of the thalamus
IGL	Intergeniculate leaflet of the lateral geniculate complex
MD	Mediodorsal nucleus of thalamus
SMT	Submedial nucleus of the thalamus
MH	Medial habenula
CL	Central lateral nucleus of the thalamus
IMD	Intermediodorsal nucleus of the thalamus
CM	Central medial nucleus of the thalamus
AD	Anterodorsal nucleus
PCN	Paracentral nucleus
PF	Parafascicular nucleus
PPT	Posterior pretectal nucleus
MRN	Midbrain reticular nucleus
RN	Red nucleus
APN	Anterior pretectal nucleus
SCm	Superior colliculus, motor related
III	Oculomotor nucleus
SNr	Substantia nigra, reticular part
MPT	Medial pretectal area
ND	Nucleus of Darkschewitsch
CUN	Cuneiform nucleus
NOT	Nucleus of the optic tract
NPC	Nucleus of the posterior commissure
OP	Olivary pretectal nucleus
VTA	Ventral tegmental area
PAG	Periaqueductal gray
VCO	Ventral cochlear nucleus
GR	Gracile nucleus
SPVC	Spinal nucleus of the trigeminal, caudal part
SPVI	Spinal nucleus of the trigeminal, interpolar part
SPVO	Spinal nucleus of the trigeminal, oral part
NTS	Nucleus of the solitary tract
DCO	Dorsal cochlear nucleus
IPN	Interpeduncular nucleus
PPN	Pedunculopontine nucleus
IF	Interfascicular nucleus raphe
SNc	Substantia nigra, compact part
CLI	Central linear nucleus raphe
DR	Dorsal nucleus raphe
SOCl	Superior olivary complex, lateral part
NLL	Nucleus of the lateral lemniscus
PSV	Principal sensory nucleus of the trigeminal
PB	Parabrachial nucleus
NLLv	Nucleus of the lateral lemniscus, ventral part
GRN	Gigantocellular reticular nucleus
MDRNv	Medullary reticular nucleus, ventral part

Abbreviation	Structure name
AMB	Nucleus ambiguus
IRN	Intermediate reticular nucleus
PRP	Nucleus prepositus
MV	Medial vestibular nucleus
LAV	Lateral vestibular nucleus
SPIV	Spinal vestibular nucleus
MARN	Magnocellular reticular nucleus
VI	Abducens nucleus
VII	Facial motor nucleus
XII	Hypoglossal nucleus
IO	Inferior olivary complex
DMX	Dorsal motor nucleus of the vagus nerve
PARN	Parvicellular reticular nucleus
LRNm	Lateral reticular nucleus, magnocellular part
PGRNl	Paragigantocellular reticular nucleus, lateral part
PRNc	Pontine reticular nucleus, caudal part
B	Barringtons nucleus
TRN	Tegmental reticular nucleus
V	Motor nucleus of trigeminal
PCG	Pontine central gray
PRNr	Pontine reticular nucleus
LC	Locus ceruleus
RPO	Nucleus raphe pontis
SLD	Sublaterodorsal nucleus
NI	Nucleus incertus
CS	Superior central nucleus raphe
RM	Nucleus raphe magnus
RO	Nucleus raphe obscurus
SIM	Simple lobule
PRM	Paramedian lobule
COPY	Copula pyramidis
PFL	Paraflocculus
FL	Flocculus
ANcr1	Crus 1
ANcr2	Crus 2
CUL4, 5	Lobules IV−V
PYR	Pyramus (VIII)
CENT2	Lobule II
CENT3	Lobule III
DN	Dentate nucleus
IP	Interposed nucleus
FN	Fastigial nucleus
